# Full-Genome Sequence of *Listeria monocytogenes* Strain H34, Isolated from a Newborn with Sepsis in Uruguay

**DOI:** 10.1128/genomeA.00544-17

**Published:** 2017-06-15

**Authors:** Francis Muchaamba, Claudia Guldimann, Taurai Tasara, María Inés Mota, Valeria Braga, Gustavo Varela, Gabriela Algorta, Jochen Klumpp, Marco Jermini, Roger Stephan

**Affiliations:** a Institute for Food Safety and Hygiene, University of Zürich, Zürich, Switzerland; b Departamento de Bacteriología y Virología, Facultad de Medicina, Instituto de Higiene, Universidad de la República, Montevideo, Uruguay; c Institute of Food, Nutrition and Health, ETH Zürich, Zürich, Switzerland; d Department of Health, Cantonal Laboratory Ticino, Division of Public Health, Bellinzona, Switzerland

## Abstract

The foodborne pathogen *Listeria monocytogenes* causes severe disease mainly in the vulnerable populations of the young, old, pregnant, and immunocompromised. Here, we present the genome sequence of *L. monocytogenes* H34, a serotype 1/2b, lineage I, sequence type 489 (ST489) strain, isolated from a neonatal sepsis case in Uruguay.

## GENOME ANNOUNCEMENT

In August 2016, 10 human cases of listeriosis were observed in Uruguay. As part of a subcluster consisting of 2 strains with similar pulsed-field gel electrophoresis (PFGE) patterns (ApaI and AscI), *L. monocytogenes* strain H34 was isolated from blood and lung cultures from a newborn with sepsis.

Serology (using antisera from Denka Seiken, Dagmersellen, Switzerland) and 7-gene multilocus sequence typing (MLST) (http://bigsdb.pasteur.fr/) identified H34 as a serotype 1/2b, lineage I, sequence type 489 (ST489), clonal complex 489 (CC489) strain. While lineage I, serotype 1/2b strains are commonly associated with human clinical cases (1), there is currently only 1 CC489 entry in the Pasteur MLST database.

For sequencing, DNA was isolated using the GenElute bacterial genomic DNA kit (Sigma, Buchs, Switzerland). The DNA was sheared to 10 kb in size and size-selected, and PacBio libraries were prepared using C4-P6 sequencing chemistry (Pacific Biosciences, Menlo Park, CA, USA). A total of 4.5 × 10^9^ bp of reads were obtained using 2 single-molecule real-time (SMRT) cells in a PacBio RSII device (Pacific Biosciences). A *de novo* assembly was performed with the HGAP_3 algorithm (2), resulting in a single contig with a 945× coverage. Genome closure was confirmed by PCR over the ends of the contig. Annotation was performed through the NCBI Prokaryotic Genome Annotation Pipeline (PGAP) (3).

The H34 genome is 2,892,474 bp in size, with a G+C content of 38.0%, and it carries no plasmids. PGAP annotated 2,823 coding sequences and 89 RNAs, and RAST (http://rast.nmpdr.org) determined the lab strain Scott A to be the closest relative to H34.

The H34 sequence was further analyzed with a focus on the virulence genes. All genes associated with the *Listeria* pathogenicity islands 1 (LIPI-1) (4) and 3 (LIPI-3) (5) were present. Compared to the *L. monocytogenes* reference strains EGDe and 10403S, two variations in H34 stand out: (i) a deletion in the *actA* coding sequence (CDS) and (ii) several single nucleotide polymorphisms within the phospholipase C (*plcB*) CDS and its 5′ untranslated region (UTR).

The H34 *actA* CDS has a 105-nucleotide (nt) in-frame deletion in the P-region of the ActA protein. While the P-region is not required for actin polymerization, it has a role in determining the length of the actin tails and the speed of bacterial motility (6), and its deletion has led to significantly reduced cellular aggregation (7). Within the remaining portion of *actA*, there is a considerable mismatch on the protein level between H34, EGDe, and 10403S. To test the hypothesis that H34 might have an aggregation defect, we performed an aggregation assay that showed no differences between H34, EGDe, and 10403S (data not shown).

There are several nonsense mutations within the first 246 bp of the *plcB* CDS, leading to either a nonfunctional or shortened PlcB protein (85 amino acids), and H34 contains the same mutations in the 5′ UTR of* plcB*, as described for strain H4 by Jiang et al. (8).

The H34 genome contains 2 incomplete prophages, as determined by Phaster (9); none of them are integrated into the* comK* CDS.

### Accession number(s).

The complete H34 genome has been deposited in the NCBI GenBank under accession number CP020774.

## References

[1] Orsi RH, den Bakker HC, Wiedmann M 2011 *Listeria monocytogenes* lineages: genomics, evolution, ecology, and phenotypic characteristics. Int J Med Microbiol 301:79–96. doi:10.1016/j.ijmm.2010.05.002. 20708964

[2] Chin CS, Alexander DH, Marks P, Klammer AA, Drake J, Heiner C, Clum A, Copeland A, Huddleston J, Eichler EE, Turner SW, Korlach J 2013 Nonhybrid, finished microbial genome assemblies from long-read SMRT sequencing data. Nat Methods 10:563–569. doi:10.1038/nmeth.2474. 23644548

[3] Tatusova T, DiCuccio M, Badretdin A, Chetvernin V, Nawrocki EP, Zaslavsky L, Lomsadze A, Pruitt KD, Borodovsky M, Ostell J 2016 NCBI Prokaryotic Genome Annotation Pipeline. Nucleic Acids Res 44:6614–6624. doi:10.1093/nar/gkw569. 27342282 PMC5001611

[4] Vázquez-Boland JA, Kuhn M, Berche P, Chakraborty T, Domínguez-Bernal G, Goebel W, González-Zorn B, Wehland J, Kreft J 2001 *Listeria* pathogenesis and molecular virulence determinants. Clin Microbiol Rev 14:584–640. doi:10.1128/CMR.14.3.584-640.2001. 11432815 PMC88991

[5] Cotter PD, Draper LA, Lawton EM, Daly KM, Groeger DS, Casey PG, Ross RP, Hill C 2008 Listeriolysin S, a novel peptide haemolysin associated with a subset of lineage I *Listeria monocytogenes*. PLoS Pathog 4:e1000144. doi:10.1371/journal.ppat.1000144. 18787690 PMC2522273

[6] Lasa I, David V, Gouin E, Marchand JB, Cossart P 1995 The amino-terminal part of ActA is critical for the actin-based motility of *Listeria monocytogenes*; the central proline-rich region acts as a stimulator. Mol Microbiol 18:425–436. doi:10.1111/j.1365-2958.1995.mmi_18030425.x. 8748027

[7] Travier L, Guadagnini S, Gouin E, Dufour A, Chenal-Francisque V, Cossart P, Olivo-Marin JC, Ghigo JM, disson O, Lecuit M 2013 ActA promotes *Listeria monocytogenes* aggregation, intestinal colonization and carriage. PLoS Pathog 9:e1003131. doi:10.1371/journal.ppat.1003131. 23382675 PMC3561219

[8] Jiang LL, Xu JJ, Chen N, Shuai JB, Fang WH 2006 Virulence phenotyping and molecular characterization of a low-pathogenicity isolate of *Listeria monocytogenes* from cow’s milk. Acta Biochim Biophys Sin 38:262–270. doi:10.1111/j.1745-7270.2006.00161.x. 16604266

[9] Arndt D, Grant JR, Marcu A, Sajed T, Pon A, Liang Y, Wishart DS 2016 PHASTER: a better, faster version of the PHAST phage search tool. Nucleic Acids Res 44:W16–W21. doi:10.1093/nar/gkw387. 27141966 PMC4987931

